# Antithrombotic Therapy in Hereditary Hemorrhagic Telangiectasia: Real-World Data from the Gemelli Hospital HHT Registry

**DOI:** 10.3390/jcm9061699

**Published:** 2020-06-02

**Authors:** Eleonora Gaetani, Fabiana Agostini, Igor Giarretta, Angelo Porfidia, Luigi Di Martino, Antonio Gasbarrini, Roberto Pola, on behalf of the Multidisciplinary Gemelli Hospital Group for HHT

**Affiliations:** 1 Multidisciplinary Gemelli Hospital Group for HHT, Fondazione Policlinico Universitario A. Gemelli IRCCS Università Cattolica del Sacro Cuore, 00168 Rome, Italy; fabiana.agostini@libero.it (F.A.); igor.giarretta@unicatt.it (I.G.); angelo.porfidia@policlinicogemelli.it (A.P.); luigidimartino7@gmail.com (L.D.M.); antonio.gasbarrini@unicatt.it (A.G.); roberto.pola@unicatt.it (R.P.); 2 Department of Translational Medicine and Surgery, Fondazione Policlinico Universitario A. Gemelli IRCCS Università Cattolica del Sacro Cuore, 00168 Rome, Italy; 3 Division of Internal Medicine, Fondazione Policlinico Universitario A. Gemelli IRCCS Università Cattolica del Sacro Cuore, 00168 Rome, Italy; 4 Department of Cardiovascular Sciences, Fondazione Policlinico Universitario A. Gemelli IRCCS Università Cattolica del Sacro Cuore, 00168 Rome, Italy

**Keywords:** Hereditary Hemorrhagic Telangiectasia, antithrombotic therapy, anticoagulants, antiplatelets, bleeding, safety

## Abstract

Although Hereditary Hemorrhagic Telangiectasia (HHT) is characterized by an overwhelming bleeding propensity, patients with this disease may also present medical conditions that require antithrombotic therapy (AT). However, precise information on indications, dosage, duration, effectiveness, and safety of AT in HHT patients is lacking. We performed a retrospective analysis of the HHT Registry of our University Hospital and found 26 patients who received AT for a total of 30 courses (19 courses of anticoagulant therapy and 11 courses of antiplatelet therapy). Indications to treatments included: atrial fibrillation, venous thrombosis and pulmonary embolism, heart valve replacement, retinal artery occlusion, secondary prevention after either stroke or myocardial infarction, and thromboprophylaxis for surgery. The total time of exposure to antiplatelet therapy was 385 months and to anticoagulant therapy 169 months. AT was generally well tolerated, with no fatal bleedings and no significant changes in hemoglobin levels. However, we found three major bleedings, with an incidence rate of 6.5 per 100 patients per year. When only patients treated with anticoagulants were considered, the incidence rate of major bleedings increased to 21.6 per 100 patients per year. Our study indicates that major bleeding may occur in HHT patients receiving AT, with a substantially increased rate in those treated with anticoagulants. Further studies are needed to fully estimate the tolerability of antithrombotic drugs in HHT.

## 1. Introduction

Hereditary Hemorrhagic Telangiectasia (HHT) is a rare autosomal dominant disease characterized by recurrent spontaneous epistaxis, visceral arteriovenous malformations (AVMs), and mucocutaneous telangiectases [1,2,3]. Five genetic types of HHT are recognized, with three being linked to particular genes. More than 80% of all cases of HHT are due to mutations in either *ENG* or *ACVRL1*, which cause HHT1 and HHT2, respectively [1,2,3]. About 2% of HHT are instead due to *SMAD4* mutations, which cause colonic polyposis in addition to HHT [1,2,3].

Although the hallmark of HHT is an overwhelming bleeding propensity, it is well known that patients with this disease are not devoid of medical conditions that require antithrombotic therapy (AT). For instance, they may have coronary artery disease (CAD), venous thromboembolism (VTE), or atrial fibrillation (AF) [4,5,6,7,8]. Additionally, they may require thromboprophylactic anticoagulation when they are hospitalized for an acute medical illness or when they undergo surgical procedures [9,10]. However, precise information on the indications, dosage, duration, and effectiveness of AT in HHT patients is lacking. Additionally, it is not clear whether subjects with HHT are able to tolerate AT, neither is it known if bleedings associated with AT are more common in some HHT phenotypes than other. Finally, no studies have been carried out to evaluate whether there are differences in AT tolerability between subjects with HHT1 or HHT2. The consequence is that clinicians are often afraid to prescribe AT to HHT patients, especially if they are not familiar with the disease. It may also happen that patients are reluctant to take these drugs, since they have been advised to avoid the use of medications that may worsen their risk of bleeding.

One of the most recent studies that evaluated the safety of AT in subjects with HHT was carried out by the European Reference Network for Rare Multisystemic Vascular Diseases (VASCERN), which retrospectively analyzed 28 HHT subjects treated with direct oral anticoagulants (DOACs) for either AF or VTE [11]. The result was that epistaxis worsened in 24 cases and in 11 cases patients had to discontinue treatment. Another recent study analyzed the RIETE Registry—which specifically consists of subjects affected by VTE—and found 23 patients with HHT (in a time frame of approximately 10 years), who presented 2 major bleedings and 6 non major bleedings during anticoagulant treatment [12]. More recently, we published the interim results of a prospective study conducted on 12 HHT subjects treated with either antiplatelet or anticoagulant therapy for various clinical indications for a mean period of approximately 6 months, who did not present any bleeding different from epistaxis while on treatment [13]. In addition, there was not epistaxis worsening and there were not significant changes in hemoglobin levels after initiation of AT [13].

Here, we carried out a retrospective analysis of the “Gemelli Hospital HHT Registry”, which contains demographic and clinical information of more than 200 subjects managed at the HHT Center of our University Hospital, with the goal to provide real-world data on indications, dosage, duration, effectiveness, and safety of AT in subjects with HHT.

## 2. Materials and Methods

We searched the Gemelli Hospital HHT Registry, which contains all the demographic and clinical information of the patients followed at the HHT Center of the ‘Fondazione Policlinico Universitario A. Gemelli IRCCS’, Rome, Italy. The creation of the Registry was approved by the Ethics Committee of the above-mentioned hospital (protocol number 2999). The time frame of the search was from 1 June 2016 (opening day of the HHT Center) to 31 December 2018.

First, we looked for subjects with a ‘definite’ diagnosis of HHT, i.e., those with genetic confirmation of the disease and/or those displaying at least 3 of the following 4 Curaçao criteria, as established in the literature [14]: (1) recurrent and spontaneous nosebleeds (epistaxis); (2) multiple telangiectases on the skin of the hands, lips, or face, or inside of the nose or mouth; (3) AVMs or telangiectases in one or more internal organs, including the lungs, brain, liver, intestine, stomach, and spinal cord; (4) a family history of HHT (i.e., first-degree relative, such as brother, sister, parent, or child, who meet these same criteria for definite HHT or has been genetically diagnosed).

Next, we identified subjects who were treated, or had been treated in the past, with AT. We registered the indications to prescription and the type and dosage of the prescribed drug. Duration of treatment and patient compliance to treatment were also registered. Since there were cases of patients that were treated with AT more than once and/or with different drugs for different indications, each course of AT was separately analyzed. We also assessed AT effectiveness and safety, evaluating whether thrombotic/ischemic events or hemorrhagic events occurred while patients were taking AT. To identify all the events, in addition to searching the Registry, we also asked patients to come to our HHT Center to undergo a detailed medical interview and fill out a questionnaire, upon signature of an informed consent. Hemorrhagic events were classified according to the criteria of the International Society on Thrombosis and Haemostasis (ISTH) [15,16]: major bleedings were defined as fatal bleedings, or symptomatic bleedings in critical areas or organs (such as intracranial, intraspinal, intraocular, retroperitoneal, intraarticular or pericardial, or intramuscular with compartment syndrome), or bleedings causing a fall in hemoglobin level of 2 g/dL or more, or leading to transfusion of two or more units of whole blood or red cells; clinically relevant non-major (CRNM) bleedings were defined as acute or subacute clinically overt bleedings that did not satisfy the criteria for major bleedings but led to hospital admission for bleeding, or physician-guided medical or surgical treatment for bleeding, or a change in AT due to bleeding; minor bleedings, with the exception of epistaxis, were defined as acute clinically overt bleedings that did not meet the criteria for either major or CRNM bleedings. We also assessed possible changes in hemoglobin levels after initiation of AT (determined by comparing hemoglobin levels measured within the three months that preceded the initiation of AT with the hemoglobin levels measured after at least 1 month of AT).

The study was approved by the Ethics Committee of the ‘Fondazione Policlinico Universitario A. Gemelli IRCSS’ (Rome, Italy) (protocol number 49901/18, ID 2329, approval date 20 December 2018).

### Statistical Analysis

The SPSS 20.0 and GraphPad Prism 7.0 software were used to perform statistical analysis. Descriptive statistics were used to outline patients’ characteristics. Results are expressed as mean ± SD or *n* (%). Incidence rate was calculated as number of bleeding events per 100 patients per year. For parametric variables, we compared means using paired samples Student’s t test. P values less than 0.05 were considered statistically significant.

## 3. Results

### 3.1. Characteristics of the Study Population

The search of the Registry led to the identification of 29 subjects with a definite diagnosis of HHT who had received AT. Of these, three were deceased at the time of study, for reasons unrelated to AT. In particular, one patient died from severe liver cirrhosis, one from progressive heart failure, and one suffered sudden cardiac death. In the first patient, the indication to AT was AF. He was treated with anticoagulants for a short period of time and then therapy was stopped due to worsening of liver failure. The second patient had AF, but he underwent left atrial appendage closure and therefore was initially treated with anticoagulants and later with aspirin. He tolerated both treatments well. In the third patient, the indication to AT was surgical VTE prophylaxis, for which he was treated with prophylactic doses of enoxaparin for 1 month, without complications.

The 26 remaining patients accepted to participate and were included in the study. The demographical and clinical characteristics of the study population are presented in Table 1. There were 14 males and 12 females with a mean age of 59.3 ± 17.0 years. Nine patients had HHT1 due to *ENG* mutations, while 15 patients had HHT2 due to mutations of *ACVRL1*, consistent with the proportion of the two types of HHT previously found in Italy [17]. A genetic confirmation of the diagnosis was not available in 2 patients, but both fulfilled the Curaçao criteria for a ‘definite’ diagnosis of HHT. All patients (100.0%) had epistaxis. Twenty-three patients (88.5%) had mucocutaneous telangiectases. There were 10 patients with pulmonary AVMs (pAVMs) (on a total of 22 who had been screened by CT scan of the chest), 5 patients with hepatic AVMs (on a total of 24 who had been screened by either abdominal ultrasound, CT scan, and/or MRI), and 2 patients with cerebral AVMs (on a total of 20 who had been screened by either CT scan and/or MRI of the brain and spinal cord). Ten patients (38.5%) had a history of GI bleeding due to the presence of GI AVMs (on a total of 15 who had been screened by endoscopic procedures).

### 3.2. Antithrombotic Therapy (AT): Indications, Type, Dosage, Duration, and Reasons for Therapy Discontinuation

The 26 HHT patients included in this study received a total of 30 AT courses. As shown in Table 2, there were 19 courses of anticoagulant therapies and 11 courses of antiplatelet therapies. The drugs used were the following: enoxaparin (6 courses), fondaparinux (4 courses), vitamin K antagonists (VKA) (4 courses), direct oral anticoagulants (DOACs) (5 courses), acetylsalicylic acid (ASA) (9 courses), clopidogrel (1 course), and indobufen (1 course).

The indications for treatment are also presented in Table 2. We found that anticoagulant drugs were prescribed to our patients for the following reasons: AF (*n* = 6), heart valve bioprosthesis (*n* = 2), pulmonary embolism (PE) (*n* = 1), cerebral vein thrombosis (CVT) (*n* = 1), superficial vein thrombosis (SVT) (*n* = 3), retinal artery occlusion (RAO) in a patient with Horton’s diseases (*n* = 1), secondary prevention after stroke in a patient with pAVMs (*n* = 1), surgical VTE prophylaxis (*n* = 4). The reasons for prescribing antiplatelet medications were: secondary prevention after stroke in patients with pAVMs (*n* = 3), secondary prevention after stroke or transient ischemic attack (TIA) (*n* = 3), secondary prevention after a myocardial infarction (MI) (*n* = 2), prevention of cardiovascular (CV) events in Horton’s disease (*n* = 1), primary CV prevention (*n* = 2).

Information regarding dosage and duration of any single AT course is presented in Table 3, along with data regarding the genetic type of HHT and the reasons for possible cessation of therapy. The total time of exposure to AT courses was 554 months. The total time of exposure to antiplatelet courses was 385 months. The total time of exposure to anticoagulant courses was 169 months. In three cases, AT was discontinued by the patient, without medical advice.

### 3.3. AT: Effectiveness and Safety

The effectiveness of anticoagulant therapy was high. In particular, patients receiving anticoagulant therapy for AF did not experience embolic stroke or systemic embolism during treatment. Patients treated with anticoagulants for PE, SVT, or CVT did not display thrombotic recurrences during treatment. There were no thrombotic complications during the two courses of anticoagulant therapy prescribed for heart valve bioprosthesis. The patient with pAVM who was treated with acenocoumarol after stroke did not experience new ischemic events during treatment. The patient with Horton’s disease that had RAO and was treated with enoxaparin lost vision on the affected eye, but therapy was started many days after onset of symptoms. Finally, patients receiving thromboprophylaxis for surgery did not have thrombotic events while on treatment.

The effectiveness of antiplatelet therapy was also high. In particular, patients with and without pAVMs who started therapy with ASA after stroke or MI did not experience new ischemic events during treatment. The same happened to the patient treated with indobufen after TIA. Finally, the two patients receiving ASA for primary CV prevention and the patient with Horton’s disease receiving clopidogrel did not experience ischemic events while on treatment.

**Figure 1:**
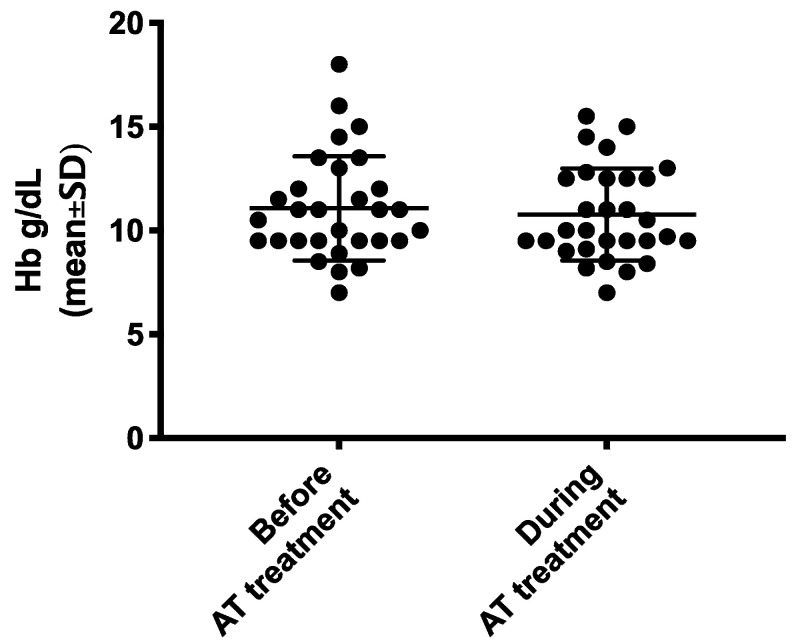
Distribution of hemoglobin (Hb) levels before and during AT.

Safety data are reported in Table 4. In total, there were three major bleedings. Of these, two were GI bleedings that occurred in the same patient during two different treatment courses with the DOAC rivaroxaban. This patient had already experienced GI bleeding before starting anticoagulation and bleeding while on rivaroxaban occurred after 6 months of treatment in one occasion and after 27 months of the treatment in another occasion. The other case of major bleeding consisted of severe hematuria in a patient treated with warfarin for AF. In the whole population, the incidence rate of major bleeding was 6.5 per 100 patients per year. In the population of patients taking anticoagulants, the incidence rate of major bleeding was 21.6 per 100 patients per year. There were no CRNM bleedings and no minor bleedings different from epistaxis. Likewise, there were no differences in the mean hemoglobin levels measured in the three months that preceded AT and those measured during AT (11.1 ± 2.5 vs. 10.8 ± 2.2 respectively, 95% CI −0.90–0.31, *p* = 0.3256) (Table 4 and Figure 1).

## 4. Discussion

This study presents data on the way AT is managed in the real-world in patients with HHT. One interesting point of discussion is the variety of medical conditions requiring AT that we found in subjects with HHT. While some of these conditions, such as secondary CV prevention, and surgical thromboprophylaxis, are relatively common for most physicians, other, such as AF, PE, CVT, RVO, Horton’s disease, and stroke in subjects with pAVMs, may be more challenging and require specialized expertise to be properly managed. For instance, in the case of AF, we found that four different anticoagulants were used, which reflects the complexity of the therapeutic choices that can be made in these patients. When the chosen anticoagulant was a DOAC, we found that a low dose was always prescribed: rivaroxaban 15 mg o.d., dabigatran 110 mg b.i.d., and apixaban 2.5 mg b.i.d. It is important to note that precise rules exist for the prescription of low doses of DOACs in patients with AF, based on age, body weight, and kidney function [18,19]. We retrospectively assessed whether the prescription of reduced doses in our patients was respectful of these rules and found that in three cases (two therapeutic courses with rivaroxaban and one therapeutic course with apixaban) prescriptions were off-label. It is known that the prescription of inappropriately low doses of DOACs may increase the risk of stroke and systemic embolism and therefore it should be avoided [20]. It is possible to speculate that the physicians that prescribed these DOACs were more concerned with the hemorrhagic, rather than the thrombotic, risk of their patients and this led to the inappropriate use of low-dose anticoagulant medications. However, it should be noted that, in the population that we analyzed, the effectiveness of these therapies were high and no thrombotic events were registered. Of course, this may be due to the limited number of patients and therefore it is not enough to justify the use of off-labeled doses of DOACs in HHT patients.

Attention should also be paid to the way secondary prevention was managed in patients with pAVMs that had an ischemic stroke. In three cases, it was prescribed an antiplatelet medication, while in another patient it was prescribed the anticoagulant acenocoumarol. This is odd, since the most recent recommendations on the medical treatment of stroke in subjects with pAVMs (produced by the British Thoracic Society) state that, as in the general population, ischemic strokes in patients with pAVM should be treated with antiplatelet agents, while anticoagulants should be used if other indications, such as AF or VTE, coexist [21].

Regarding safety, our study shows that 90% of AT courses was completed without the occurrence of significant hemorrhagic events and that only 10% of them was complicated by episodes of major bleeding. Importantly, there were no episodes of fatal bleeding. Additionally, hemoglobin levels, which are an objective outcome, did not change between before and after starting AT. However, when the incidence of major bleeding was calculated, based on time of exposure to AT, it resulted to be as high as 6.5/100 patients/year. Importantly, the incidence of major bleeding raised to 21.6/100 patients/year when only patients taking anticoagulants were considered. On the other hand, there were no episodes of major bleeding among HHT subjects treated with antiplatelet medications, although the total exposure time to antiplatelet therapy (385 months) was approximately twice the total exposure time to anticoagulant therapy (169 months).

As already mentioned in the Introduction section, two other studies have recently evaluated whether HHT subjects tolerate AT [11,12]. These studies and ours have many similarities. First, they include a similar number of HHT patients: 28 in the study by Shovlin et al. [11], 23 in the study by Riera-Mestre et al. [12], and 26 in our study. Additionally, all these studies have a retrospective design: Shovlin et al. utilized the databases of VASCERN-participating centers [11], Riera-Mestre et al. analyzed the RIETE Registry [12], and we studied the database of the HHT Center of our University Hospital. However, there are also important differences between our study and the other two. For instance, in the study of Riera-Mestre et al., the authors were not able to retrospectively assess Curaçao criteria and/or collect genetic data for all patients [12]. In contrast, in our study, all patients had a ‘definite’ diagnosis of HHT and a genetic confirmation of the disease was present in 24 out of 26 patients. Another difference is that Shovlin et al. and Riera-Mestre et al. only studied subjects treated with anticoagulants (and only with DOACs in the case of Shovlin et al.) [11,12], while we included individuals treated with antiplatelet medications. Additionally, the indications to treatment were different between these three studies. In Shovlin’s study, all patients were affected by either AF or VTE [11], while Riera-Mestre et al. only evaluated subjects with VTE [12]. In contrast, the patients investigated in our study had more heterogeneous indications to AT, which included stroke, MI, thromboprophylaxis, and other diseases. Regarding the results of these studies, Shovlin et al. found that epistaxis worsened in 24 cases and in 11 cases patients had to discontinue treatment, on a total of 28 patients [11]. However, all their subjects were treated exclusively with DOACs. In addition, they did not provide precise information on the medical regimen followed by patients and did not carry out a distinction between bleedings, in terms of severity [11]. On the other hand, Riera-Mestre et al. found two major bleedings and six non major bleedings during anticoagulant treatment, on a total of 23 patients [12]. Precise information on the medical regimen followed by patients was not provided in this case either. Numerically speaking, our results (three major bleedings on a total of 26 patients who received a total of 30 AT courses) appear similar to those reported by Riera-Mestre et al. However, as mentioned above, our population is different, because it did not only include subjects with VTE who were treated with anticoagulants. Therefore, no direct comparisons can be made.

Other important reports available in the literature on this topic are those published by Edwards et al. in 2012 and Devlin et al. in 2013 [22,23]. A main difference between these two studies and ours is that we were able to distinguish between major, CRNM, and minor bleedings, thus providing data not only on the number of hemorrhagic complications, but also on their severity and clinical impact, while this was not done in the study by Devlin et al. [23]. Additionally, the subjects studied by Devlin et al. did not always have a ‘definite’ diagnosis of HHT [23] and those investigated by Edwards et al. had lower rates of epistaxis, GI bleeding, anemia, and visceral AVMs compared to our patients [22].

Our study has some limitations. It has a small sample size and a retrospective nature. It is possible that some HHT subjects did not receive a prescription of AT despite the presence of a clinical indication, and this might be a selection bias of our study. Additionally, the difference that we saw in the safety of antiplatelet drugs compared to anticoagulant therapies might depend on the small sample size, which also did not allow us to make distinctions between different types of antiplatelet and anticoagulant medications. It is also possible that HHT subjects requiring anticoagulation are different from those requiring antiplatelet therapy and that the increased rate of bleeding that we observed in subjects treated with DOACs depends on the intrinsic characteristics of the patients rather than on the drugs used. Finally, the small sample size did not allow us to investigate whether HHT1 and HHT2 patients have different tolerability to AT, which is a very intriguing issue that deserves further investigation.

In conclusion, our study presents real-world data on HHT subjects treated with AT, providing novel information on the way antiplatelet and anticoagulant medications are used in this unique population, which has an intrinsically high hemorrhagic risk. Our results indicate that major bleeding may occur in HHT patients receiving AT, especially those treated with anticoagulants. Further studies are needed to better define the optimal use of these medications and fully assess the safety profile of various types of AT agents in HHT patients.

## Figures and Tables

**Table 1:** Characteristics of the study population.

Mean age (years ± SD)	59.3 ± 17.0
Gender (male/female ratio)	14/12
HHT1/HHT2/Clinical diagnosis (*n*)	9/15/2
Epistaxis (*n*/total)	26/26
Mucocutaneous telangiectases (*n*/total)	23/26
Family history of HHT (*n*/total)	24/26
Pulmonary AVMs (*n*/screened)	10/22
Hepatic AVMs (*n*/screened)	5/24
Cerebral AVMs (*n*/screened)	2/20
Gastrointestinal AVMs (*n*/screened)	10/15
Previous gastrointestinal bleeding (*n*/total)	10/26

AT: antithrombotic therapy; HHT: Hereditary Hemorrhagic Telangiectasia; AVMs: arteriovenous malformations; SD: standard deviation.

**Table 2:** Type and number of AT courses and reasons for prescription.

**Anticoagulant drug**	**Number of therapeutic courses (*n* = 19)**
enoxaparin	6
fondaparinux	4
VKA	4
DOAC	5
**Antiplatelet drug**	**Number of therapeutic courses (*n* = 11)**
ASA (number of courses)	9
clopidogrel (number of courses)	1
indobufen (number of courses)	1
**Reasons why anticoagulant therapy was prescribed**	
AF	6
Heart valve bioprosthesis	2
PE	1
CVT	1
SVT	3
RAO in a patient with Horton’s disease	1
Secondary prevention after stroke/TIA in a patient with pAVMs	1
Surgical VTE prophylaxis	4
**Reasons why antiplatelet therapy was prescribed**	
Secondary prevention after stroke in patients with pAVMs	3
Secondary prevention after stroke/TIA	3
Secondary prevention after MI	2
Horton’s disease	1
Primary CV prevention	2

AT: antithrombotic therapy; VKA: vitamin K antagonist; DOAC: direct oral anticoagulant; ASA: acetylsalicylic acid; AF: atrial fibrillation; PE: pulmonary embolism; CVT: cerebral vein thrombosis; SVT: superficial vein thrombosis; VTE: venous thromboembolism; TIA: transient ischemic attack; pAVMs: pulmonary arteriovenous malformations; RAO: retinal artery occlusion; MI: myocardial infarction; CV: cardiovascular.

**Table 3:** Dosage, duration of treatment, compliance to AT, and reasons for therapy discontinuation.

Drug	Genetic Mutation	Indication for Treatment	Dosage Prescribed	Treatment Duration	Ongoing Treatment at the Time of Study	Reason for Therapy Cessation	Therapy Cessation Decided by Doctor or Patient
warfarin	*ACVRL1* (HHT2)	AF	variable, based on INR	6 months	No	hematuria	doctor
rivaroxaban	*ACVRL1* (HHT2)	AF	15 mg o.d.	6 months	No	GI bleeding	doctor
rivaroxaban	*ACVRL1* (HHT2)	AF	15 mg o.d.	27 months	No	GI bleeding	doctor
rivaroxaban	*ACVRL1* (HHT2)	AF	15 mg o.d.	13 months	Yes	---	---
dabigatran	*ACVRL1* (HHT2)	AF	110 mg b.i.d.	21 months	Yes	---	---
apixaban	*ACVRL1* (HHT2)	AF	2.5 mg b.i.d.	20 months	Yes	---	---
acenocoumarol	*ENG* (HHT1)	stroke in pAVM	variable, based on INR	46 months	Yes	---	---
enoxaparin	*ENG* (HHT1)	SVT	4000 U b.i.d.	1 month	No	completion of treatment	doctor
enoxaparin	*ACVRL1* (HHT2)	SVT	4000 U b.i.d.	1 month	No	completion of treatment	doctor
fondaparinux	*ACVRL1* (HHT2)	SVT	2.5 mg o.d.	1 month	No	completion of treatment	doctor
warfarin	*ENG* (HHT1)	heart valve bioprosthesis	variable, based on INR	3 months	No	completion of treatment	doctor
fondaparinux	Clinical diagnosis	heart valve bioprosthesis	2.5 mg o.d.	3 months	No	completion of treatment	doctor
warfarin	*ACVRL1* (HHT2)	PE	variable, based on INR	1 month	No	shift to DOAC	doctor
enoxaparin	*ENG* (HHT1)	RAO in Horton’s disease	6000 U b.i.d	5 months	No	completion of treatment	doctor
fondaparinux	*ENG* (HHT1)	CVT	5 mg o.d.	6 months	No	completion of treatment	doctor
enoxaparin	*ACVRL1* (HHT2)	surgical VTE prophylaxis	4000 U o.d.	1 month	No	completion of treatment	doctor
enoxaparin	*ACVRL1* (HHT2)	surgical VTE prophylaxis	4000 U o.d.	3 months	No	completion of treatment	doctor
enoxaparin	*ACVRL1* (HHT2)	surgical VTE prophylaxis	4000 U o.d.	4 months	No	completion of treatment	doctor
enoxaparin	*ACVRL1* (HHT2)	surgical VTE prophylaxis	4000 U o.d.	1 month	No	completion of treatment	doctor
ASA	*ENG* (HHT1)	stroke in pAVM	100 mg o.d.	43 months	No	worsening of epistaxis	patient
ASA	*ENG* (HHT1)	stroke in pAVM	100 mg o.d.	72 months	No	pAVM embolization	doctor
ASA	*ENG* (HHT1)	stroke in pAVM	100 mg o.d.	14 months	Yes	---	---
ASA	Clinical diagnosis	Stroke	100 mg o.d.	30 months	No	worsening of epistaxis	patient
ASA	*ACVRL1* (HHT2)	Stroke	100 mg o.d.	5 months	Yes	---	---
indobufen	*ENG* (HHT1)	TIA	200 mg o.d.	21 months	Yes	---	---
ASA	*ACVRL1* (HHT2)	secondary prevention after MI	100 mg o.d.	94 months	Yes	---	---
ASA	*ACVRL1* (HHT2)	secondary prevention after MI	100 mg o.d.	50 months	Yes	---	---
ASA	*ACVRL1* (HHT2)	primary CV prevention	100 mg o.d.	24 months	No	worsening of epistaxis	patient
ASA	*ACVRL1* (HHT2)	primary CV prevention	100 mg o.d.	18 months	No	medical decision	doctor
clopidogrel	*ENG* (HHT1)	Horton’s disease	75 mg o.d.	14 months	Yes	---	---

AT: antithrombotic therapy; AF: atrial fibrillation; INR: international normalized ratio; o.d.: once daily; GI: gastrointestinal; b.i.d.: bis in die; pAVM: pulmonary arteriovenous malformation; SVT: superficial vein thrombosis; PE: pulmonary embolism; DOAC: direct oral anticoagulant; RAO: retinal artery occlusion; CVT: cerebral vein thrombosis; VTE: venous thromboembolism; ASA: acetylsalicylic acid; TIA: transient ischemic attack; MI: myocardial infarction; CV: cardiovascular.

**Table 4:** Bleeding complications during AT.

Minor bleedings different from epistaxis (*n*/total of AT courses)	0/30
CRNM bleedings (*n*/total of AT courses)	0/30
Major bleedings (*n*/total of AT courses)	3/30
- in subjects taking anticoagulants (*n*/total of anticoagulant courses)	3/19
- in subjects taking antiplatelets (*n*/total of antiplatelet courses)	0/11
Hb levels (g/dL) during AT versus prior to AT (mean ± SD)	10.8 ± 2.2 vs. 11.1 ± 2.5 (95% CI −0.90–0.31) *p* = 0.3256

AT: antithrombotic therapy; CRNM: clinically relevant non-major; Hb: hemoglobin; SD: standard deviation.
